# Preoperative rapid growth of inferior vena cava tumor thrombus in renal cell carcinoma

**DOI:** 10.1002/iju5.12709

**Published:** 2024-02-20

**Authors:** Akifumi Katsu, Masato Yanagi, Masato Yoshioka, Norio Motoda, Hideyuki Takata, Hiroyoshi Kono, Ryoji Kimata, Tsutomu Hamasaki, Yukihiro Kondo

**Affiliations:** ^1^ Department of Urology Nippon Medical School Musashikosugi Hospital 1‐383, Kosugimachi 211‐8533 Kanagawa Japan; ^2^ Department of Surgery Nippon Medical School Musashikosugi Hospital Kawasaki Kanagawa Japan; ^3^ Department of Pathology Nippon Medical School Musashikosugi Hospital Kawasaki Kanagawa Japan; ^4^ Department of Urology Nippon Medical School Hospital Tokyo Japan

**Keywords:** inferior vena cava, renal cell carcinoma, tumor thrombus

## Abstract

**Introduction:**

We present the case of a rapidly growing inferior vena cava tumor thrombus in renal cell carcinoma.

**Case presentation:**

We present a case of a 66‐year‐old woman with right renal cell carcinoma with a tumor thrombus extending 2 cm into the inferior vena cava on an initial Imaging. Radical surgery was performed 6 weeks after the first visit. Intraoperatively, the tumor thrombus was confirmed to have grown near the diaphragm. The tumor was resected using an inferior vena cava clamping just below the diaphragm. The tumor thrombus and renal cell carcinoma were completely removed. There was no recurrence 6 months postoperatively.

**Conclusion:**

Inferior vena cava tumor thrombus in renal cell carcinoma can grow in a short period, suggesting that preoperative imaging evaluation at the appropriate time is important. Once inferior vena cava tumor thrombus of renal cell carcinoma occurs, surgery should not be delayed unless there is an urgent reason.

**Abbreviations & Acronyms**
ALT: alanine aminotransferase
AST: aspartate aminotransferase
CRP: C‐reactive protein
IVC: inferior vena cava
MRI: magnetic resonance imaging
N.A.: not available
NLR: neutrophil/lymphocyte ratio
POD: postoperative day
RCC: renal cell carcinoma
TT: tumor thrombus

**Keynote message**

Preoperative imaging evaluation of inferior vena cava tumor thrombus at the appropriate time is important because they can grow in a short period of time. Once renal cell carcinoma with an inferior vena cava tumor thrombus is diagnosed, surgery should not be delayed unless there is an urgent reason.

## Introduction

At diagnosis, 4% to 10% of RCC have an IVC TT.^1^, ^2^ Surgery for RCC with IVC TT is a highly invasive procedure because of the need to treat the IVC and a large amount of bleeding due to well‐developed collateral vessels. The mortality and complication rates are approximately 1%–10% and 18%–47%, respectively, depending on the level of IVC TT.^2^, ^3^ However, cases without metastasis, long‐term survival can be expected with radical surgery.^2^ Therefore, radical resection of RCC, including IVC TT, is the standard treatment for nonmetastatic RCC with IVC TT. The surgical difficulty of IVC TT surgery varies depending on the level of the TT: Level 0 IVC TT may be treated with simple renal vein ligation, whereas level 4 IVC TT requires surgery in collaboration with different surgical teams.^1^ Especially, level 4 IVC TT generally requires more extensive surgical procedures including thoracotomy. Therefore, a preoperative diagnosis of the level of IVC TT is important.

We describe a case in which level 2 IVC TT on preoperative MRI rapidly increased to level 3 at the time of surgery 6 weeks after the MRI.

## Case presentation

**Fig. 1:**
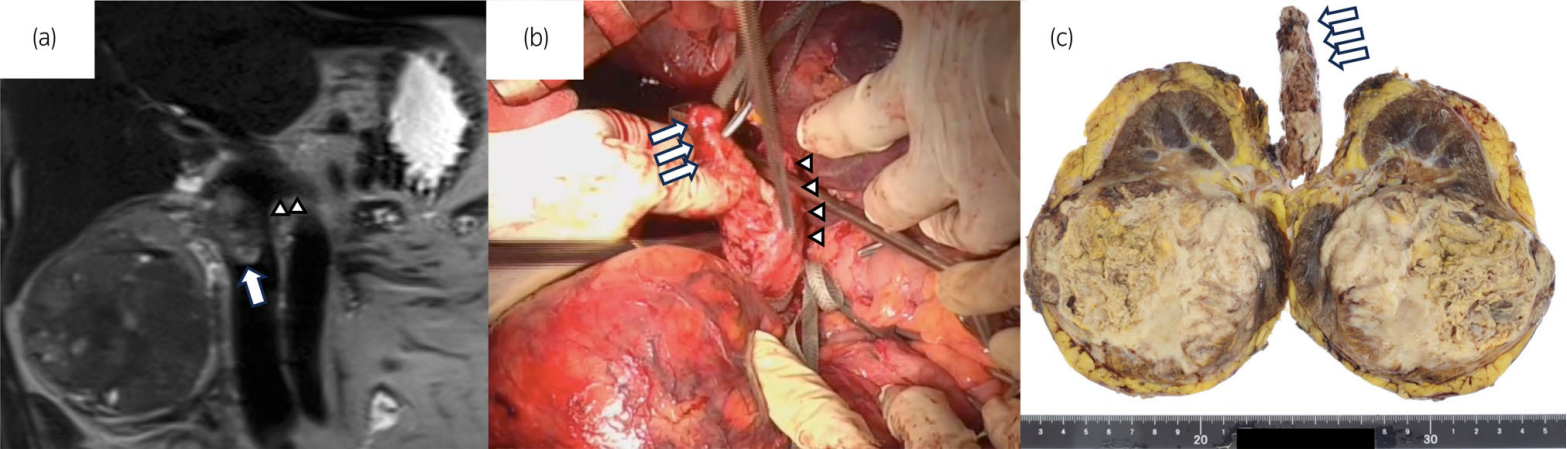
Findings of IVC TT. (a) Preoperative MRI finding: White arrows show IVC TT of the right RCC and white arrowheads show the left renal vein. (b) Intraoperative finding: White arrows show IVC TT of the right RCC and white arrowheads show the IVC. (c) Resected specimen: White arrows show IVC TT.

**Fig. 2:**
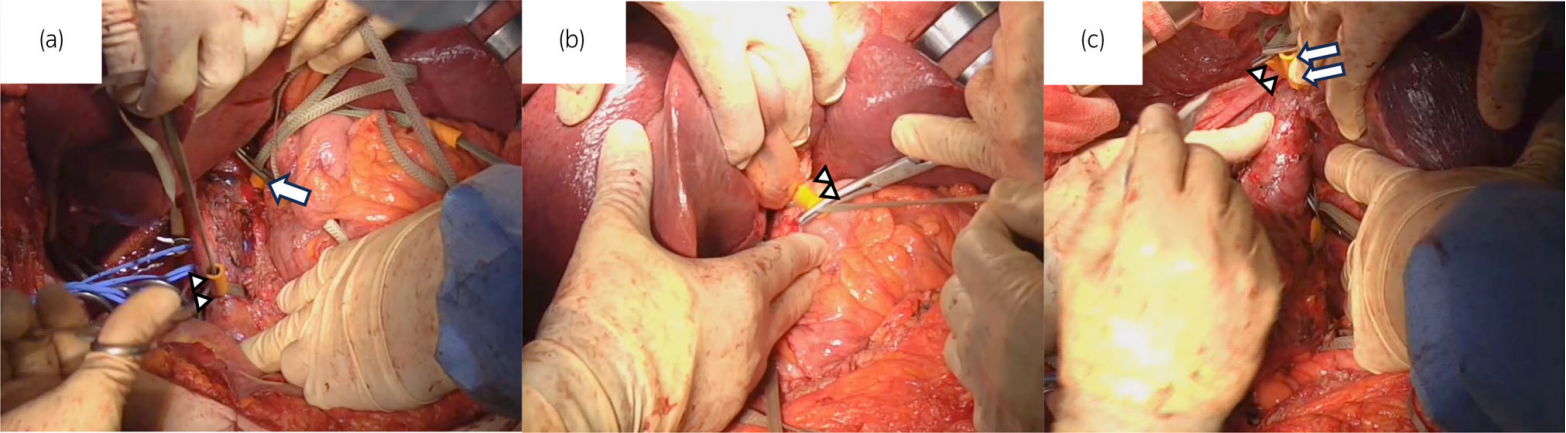
Intraoperative finding. (a) A white arrow shows left renal vein clamping. White arrowheads show IVC clamping below IVC TT. (b) White arrowheads show hepatoduodenal ligament clamping. (c) White arrows show hepatic vein clamping. White arrowheads show IVC clamping just below the diaphragm.

**Fig. 3:**
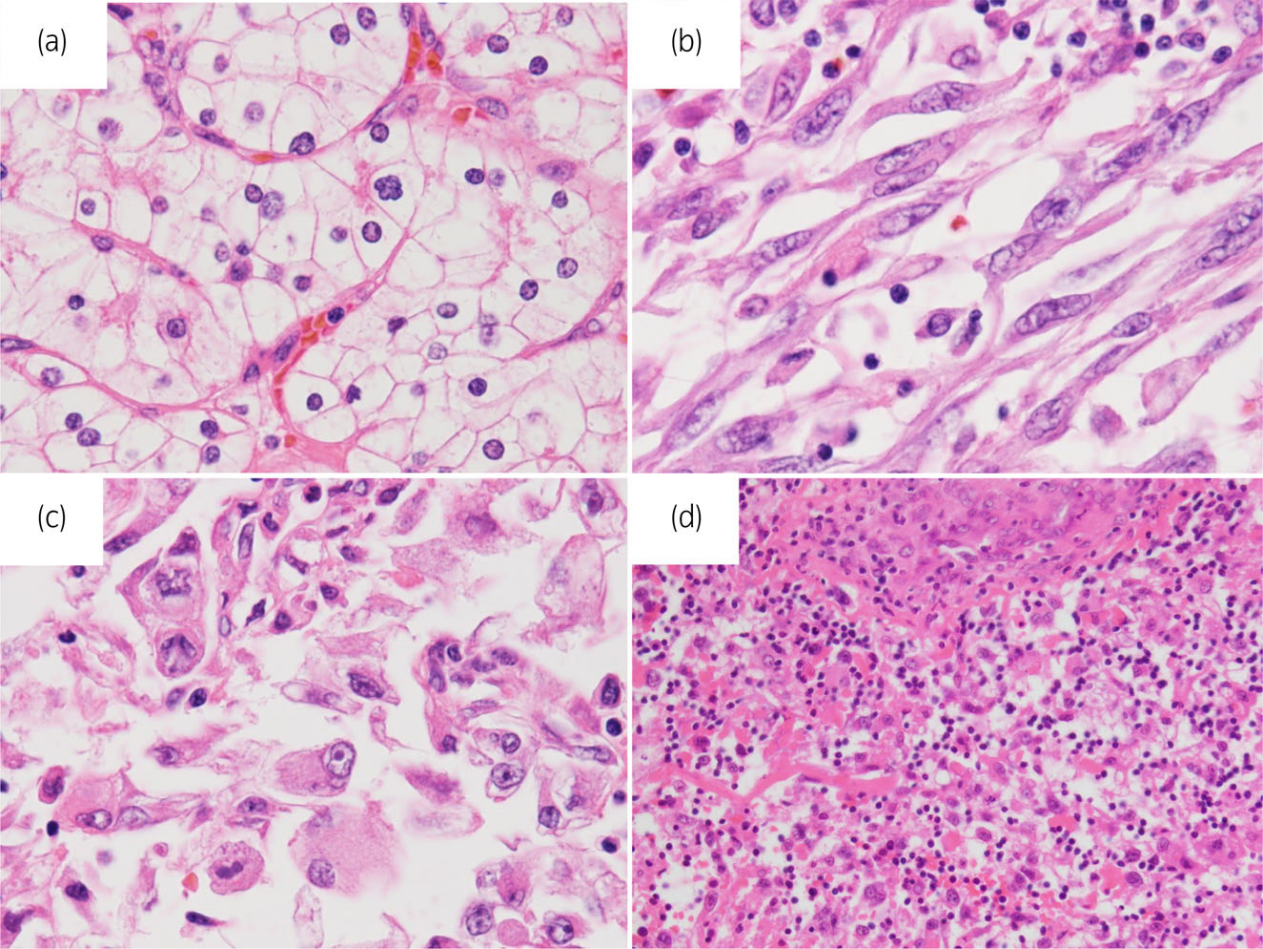
Pathological findings of the tumor. (a) The part of conventional clear cell RCC that shows a nested pattern composed of cells with clear cytoplasm and ovoid nuclei is observed (WHO/ISUP grade 2, Furhman grade 2). (b) The part of RCC with sarcomatoid change is composed of elongated spindle cells with prominent nucleoli arranged in intersecting fascicles (WHO/ISUP grade 4, Furhman grade 4). (c) Rhabdoid change, representing eccentric nuclei and abundant eosinophilic cytoplasm, is confirmed in a part of the renal carcinoma. (d) In part, IVC TT contains RCC cells.

A 66‐year‐old woman presented with a right RCC of 90 mm with a TT extending 2 cm into the IVC (Fig. 1a). Her chief complaint was mild palpitations and her hemoglobin was 7.2 g/dL. The levels of blood C‐reactive protein, white blood cell, and neutrophil were 11.36 mg/dL, 9550/μL, and 7620/μL. Neutrophil/lymphocyte ratio was 6.74. The patient was diagnosed with RCC with level 2 IVC TT (cT3bN0M0). Preoperative MRI revealed that the IVC TT had not reached the level of the left renal vein (Fig. 1a). Radical surgery was performed 6 weeks after the preoperative MRI. Intraoperative ultrasonography confirmed the status of the IVC TT, which showed an IVC TT extending just below the diaphragm. Therefore, this required IVC clamping just below the diaphragm, left renal vein clamping, and hepatoduodenal ligament clamping (Fig. 2). When the IVC was incised, the tumor was entirely adhered to it (Fig. 1b). Most tumor thrombi were removed along with the kidneys (Fig. 1c). The remaining tumor, which was adhered to the IVC, was grossly completely removed using a sharp spoon. Subsequently, the IVC was sutured for repair. During the course of suturing, the IVC clamp was changed to a caudal clamp of the hepatic vein, and hepatoduodenal ligament was de‐clamped early. Hepatoduodenal ligament clamping time was 37 min, and total clamping time was 66 min. The operative time was 585 min, and the blood loss was 2130 mL. Hepatic dysfunction (GOT 685, GPT 355) was observed immediately after surgery but improved on postoperative day 5 (Table 1). No other serious postoperative complications occurred, and the patient was discharged on postoperative day 15. The pathological diagnosis was clear cell carcinoma with sarcomatoid and rhabdoid changes (Fuhrman grade 4, WHO/ISUP grade 4) (Fig. 3a–c), pT3b. IVC TT mainly consists of fibrin clots and necrotic tissues. In part, IVC TT contains RCC cells (Fig. 3d). Because of the high risk of recurrence based on the pathological diagnosis, we recommended adjuvant therapy with pembrolizumab for the patient to reduce the risk of recurrence. However, she refused it. No recurrence was observed at 6 months postoperatively.

**Table 1:** Laboratory results for the patient

	Preoperatively	POD0	POD1	POD2	POD5	POD10
AST (Normal 7–38 IU/L)	30	684	465	128	29	20
ALT (5–45 IU/L)	28	355	278	128	29	21
Cre (<1.0 mg/dL)	1.71	1.73	2.09	2.51	2.05	1.66
D, dimer (<1.0 μg/mL)	N.A.	N.A.	7.88	6.24	14.39	7.74

## Discussion

Preoperative imaging evaluation of the IVC TT is important because it is significantly associated with surgical procedures. Multidetector CT and MRI are reportedly useful in evaluating the level of IVC TT.^1^ On the other hand, ultrasonography is noninvasive and commonly used in the evaluation of RCC, but its accuracy is highly dependent on the skill of the sonographer and the location of the IVC TT.^1^ It should be noted that the sensitivity of ultrasonography below the level of the hepatic vein is not high (68%).^1^ Rapid growth of IVC TT in the short preoperative period has been reported.^4^ Failure to recognize the rapid expansion of IVC TT ahead of surgery can have catastrophic repercussions, such as an abrupt and drastic change in surgical method, or, even worse, the decision to abort the surgery while it is being performed. Therefore, the timing of preoperative imaging is important.

Froehner *et al*. reported a case in which levels 1–2 IVC TT became level 3 within 1 month prior to surgery. The pathological diagnosis was clear cell carcinoma, pT3b, Fuhrman grade 4.^4^ In the present case, the pathological diagnosis was pT3b, clear cell carcinoma, Fuhrman grade 4. Fuhrman grade 4 might have the potential to accelerate the rapid progression of IVC TT. However, the preoperative identification of Fuhrman grade 4 is difficult. All RCC cases with IVC TT require preoperative follow‐up imaging at an appropriate time to allow for planning the treatment, especially when rapid growth of the tumor embolus occurs prior to surgery.^1^ Almatari *et al*. suggested that it is preferable for surgeons to perform weekly imaging evaluations prior to surgery to detect unexpected growth of IVC TT.^1^ However, frequent imaging examinations are time‐consuming and place an economic burden on the patient. Our institution decided to perform follow‐up ultrasonography for IVC TT evaluation 2 weeks, 1 week, and 1 day prior to surgery.

On the other hand, our surgical procedure was not ideal. It is generally believed that hepatoduodenal ligament clamping should be limited to 15 min or less to preventing intestinal congestion and hepatic ischemia. However, in the present case, hepatoduodenal ligament clamping lasted as long as 37 min due to unexpected tumor adhesion to the IVC. Ideally, the surgeon should estimate the time required for thrombectomy before opening the IVC and consider preparing partial extracorporeal circulation, if necessary. In addition, inferior vena cavectomy might have been also considered an option in this case, in which the IVC is filled with TT. We did not select the inferior vena cavectomy because we considered the collateral vessels underdeveloped, especially the collateral vessels of the left renal vein. The lack of clinical symptoms, such as leg edema, despite the rapid growth of the IVC TT resulted in the conclusion that the obstruction of the IVC was partial. However, evaluation of the presence of sufficient collateral vessels, including whether clamping of the left renal vein would cause renal enlargement, should have been performed.

An accurate preoperative evaluation of the level of the IVC TT is required because an inaccurate preoperative evaluation of the level of the IVC TT could have a fatal negative impact on the surgery. Once the IVC TT is diagnosed, it is important to perform surgery as soon as possible. However, in real‐world practice, the waiting time for surgery varies among institutions. Preoperative systemic therapy with tyrosine kinase inhibitors, such as axitinib, pazopanib, and sunitinib, has been reported to be effective in reducing or preventing the progression of the IVC TT.^5^ When the waiting time for surgery is long, preoperative systemic therapy should be considered.

## Author contributions

Akifumi Katsu: Conceptualization; data curation; methodology; writing – original draft. Masato Yanagi: Conceptualization; project administration; writing – original draft; writing – review and editing. Masato Yoshioka: Data curation. Norio Motoda: Data curation. Hideyuki Takata: Data curation. Hiroyoshi Kono: Data curation. Ryoji Kimata: Data curation. Tsutomu Hamasaki: Data curation. Yukihiro Kondo: Supervision; writing – original draft; writing – review and editing.

## Conflict of interest

The authors declare no conflict of interest.

## Approval of the research protocol by an Institutional Reviewer Board

The protocol for this research project has been approved by a suitably constituted Ethics committee of Nippon Medical School Hospital (Approved number 29‐11‐861) and it conforms to the provisions of the Declaration of Helsinki.

## Informed consent

Written informed consent was obtained from the patient to publish this case report and accompanying images.

## Registry and the Registration No. of the study/trial

Not applicable.

## Data Availability

The data sets analyzed in the current study are available from the corresponding author upon reasonable request.
